# Native Rodent Species Are Unlikely Sources of Infection for *Leishmania (Viannia) braziliensis* along the Transoceanic Highway in Madre de Dios, Peru

**DOI:** 10.1371/journal.pone.0103358

**Published:** 2014-07-25

**Authors:** Lisa A. Shender, Maxy De Los Santos, Joel M. Montgomery, Patricia A. Conrad, Bruno M. Ghersi, Hugo Razuri, Andres G. Lescano, Jonna A. K. Mazet

**Affiliations:** 1 Wildlife Health Center, One Health Institute, School of Veterinary Medicine, University of California Davis, Davis, California, United States of America; 2 Parasitology Department, U.S. Naval Medical Research Unit 6, Lima, Peru; 3 Emerging Infections Department, U.S. Naval Medical Research Unit 6, Lima, Peru; Royal Tropical Institute, Netherlands

## Abstract

An estimated 2.3 million disability-adjusted life years are lost globally from leishmaniasis. In Peru's Amazon region, the department of Madre de Dios (MDD) rises above the rest of the country in terms of the annual incidence rates of human leishmaniasis. *Leishmania* (*Viannia*) *braziliensis* is the species most frequently responsible for the form of disease that results in tissue destruction of the nose and mouth. However, essentially nothing is known regarding the reservoirs of this vector-borne, zoonotic parasite in MDD. Wild rodents have been suspected, or proven, to be reservoirs of several *Leishmania* spp. in various ecosystems and countries. Additionally, people who live or work in forested terrain, especially those who are not regionally local and whose immune systems are thus naïve to the parasite, are at most risk for contracting *L.* (*V.*) *braziliensis*. Hence, the objective of this study was to collect tissues from wild rodents captured at several study sites along the Amazonian segment of the newly constructed Transoceanic Highway and to use molecular laboratory techniques to analyze samples for the presence of *Leishmania* parasites. Liver tissues were tested via polymerase chain reaction from a total of 217 rodents; bone marrow and skin biopsies (ear and tail) were also tested from a subset of these same animals. The most numerous rodent species captured and tested were *Oligoryzomys microtis* (40.7%), *Hylaeamys perenensis* (15.7%), and *Proechimys* spp. (12%). All samples were negative for *Leishmania*, implying that although incidental infections may occur, these abundant rodent species are unlikely to serve as primary reservoirs of *L.* (*V*.) *braziliensis* along the Transoceanic Highway in MDD. Therefore, although these rodent species may persist and even thrive in moderately altered landscapes, we did not find any evidence to suggest they pose a risk for *L. (V.) braziliensis* transmission to human inhabitants in this highly prevalent region.

## Introduction

An estimated 2.3 million disability-adjusted life years are lost globally from leishmaniasis [1]. In the untreated mucocutaneous form of disease (MCL), highly destructive lesions of the nose and mouth may lead to difficulties in breathing and eating, as well as social ostracization. This severely disfiguring disease is most frequently caused by *Leishmania* (*Viannia*) *braziliensis*, a vector-borne protozoan parasite. Along with a suite of other *Leishmania* species, this parasite also causes cutaneous leishmaniasis (CL), which while less severe than MCL, can still result in serious consequences. Secondary bacterial and fungal infections are common with leishmaniasis [2], and although skin lesions may eventually heal in months to years, *L.* (*V.*) *braziliensis* organisms that have been apparently inactive can cause metastatic lesions to the face decades later via spread through the bloodstream [3].

According to the World Health Organization, at least 88 nations are affected by leishmaniais; however, just seven countries, Peru among them, account for 90% of the global annual human cases [1]. Of the two *Leishmania* subgenera, *L*. (*Viannia*) predominates in Peru and is represented by primarily four species: *L. lainsoni*, *L. braziliensis*, *L. guyanensis*, and *L. peruviana* [4], [5]. Recent reports suggest that two additional species (*L. colombiensis* and *L. panamensis*) may now be present in Peru [5]. Within the subgenus *L*. (*Leishmania*), only *L. amazonensis* species is present.

**Figure 1:**
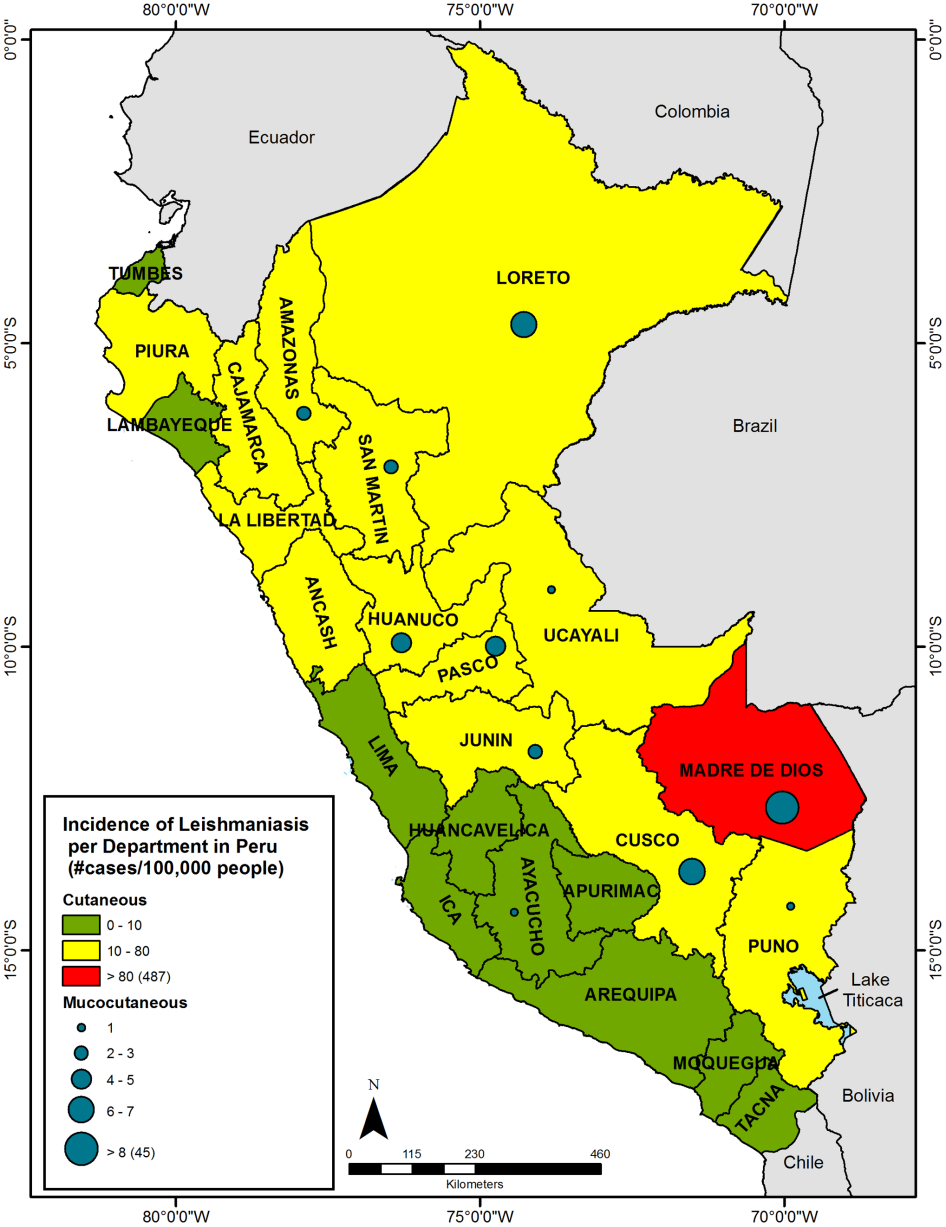
Human leishmaniasis incidence in Peru, by Department (2012). Map of Peruvian Departments indicating the 2012 incidence rates of human cutaneous (CL) and mucocutaneous (MCL) leishmaniasis cases, where incidence  =  [(number of probable and confirmed laboratory cases in epidemiological reporting week #51)/(2012 estimated population)]*(100,000 people). Madre de Dios had an incidence of 487 and 45 cases for CL and MCL, respectively. The Department of Callao had no reported cases and is excluded on this map. Numerator data was obtained from the Ministerio de Salud [7], [60] and population denominator data obtained from the Instituto Nacional de Estadística e Informática (INEI) [8].

The Peruvian department of Madre de Dios (MDD) is located in the country's Amazon region and borders both Brazil and Bolivia. This department has the lowest human population density, with an average of just 1.3 inhabitants/square kilometer in 2009 [6], yet has the highest incidence rate of leishmaniasis, with 487 cutaneous cases/100,000 people reported up through week 51 of 2012 (Figure 1)[7], [8]. In reality, the true incidence rate in MDD is probably much higher, in part due to under-reporting associated with the difficulty of reaching medical attention from remote and impoverished communities, as well as the likely possibility that infections acquired in MDD are treated and recorded as cases in neighboring departments as people migrate between regions for employment opportunities [9]. In addition to environmental factors (e.g. habitat and climate), several other factors likely contribute to the high number of cases in MDD, including human activities that bring people into contact with vectors, such as road construction, extractive industries (e.g. mining and logging), agriculture, and an influx of naïve people migrating to this region from other parts of the country [6]. Leishmaniasis has long been recognized as a serious health problem in the Peruvian Amazon. Yet, with the exception of recently published work on *Leishmania* in sand fly vectors in MDD [10], little attention has been given to the investigation of the disease ecology of *L*. (*V*.) *braziliensis* in MDD. Moreover, the wildlife reservoirs in this region are entirely unknown.

Much of the limited knowledge of potential reservoirs of *L*. (*V*.) *braziliensis* comes from field studies conducted in Brazil and Venezuela, where researchers have conflicting opinions on whether domestic animals serve as reservoirs. In the mid-1980s, the isolation of *L*. (*V*.) *braziliensis* from dogs and donkeys in communities with concurrent outbreaks of human leishmaniasis led some researchers to believe that these domestic animals may serve as reservoirs in certain settings [11]. Others have refuted this idea, stating that epidemiological evidence does not support domestic species as reservoirs, as domestic animals are largely absent from some Amazon areas where *L.* (*V.*) *braziliensis* is highly endemic, leading some to speculate that wildlife are likely more important in the maintenance and transmission of the disease [12]. However, molecular research on *L*. (*V*.) *braziliensis* suggests that these two lines of thought may not be at complete odds. Cupolillo et al. [13] found that dogs and people were infected with an identical genotype of *L*. (*V*.) *braziliensis* in an urban area along the Brazilian Atlantic coast. In contrast, parasites isolated from the Brazilian Amazon forested areas demonstrated much higher genetic diversity. The authors reasoned that their findings could be reflective of the wide variety of hosts and vectors present in the Brazilian Amazon, and that when introduced into an urban area, *L*. (*V*.) *braziliensis* is restricted to peridomestic vectors and domestic vertebrate hosts, decreasing the parasite's local genetic diversity.

Although researchers do not agree on the role of domestic animals as reservoirs of *L.* (*V*.) *braziliensis*, there is a general consensus that wildlife hosts exist and remain unidentified. While the sylvatic transmission cycle of leishmaniasis has been well established for some *Leishmania* species, the characterization of wildlife reservoirs of *L*. (*V*.) *braziliensis* has continued to elude scientists for decades [14]. In more recent years, limited data have supported the notion that rodents play a role in the sylvatic transmission cycle of *L.* (*V*.) *braziliensis* [12], [15], just as many other *Leishmania* species have been detected in rodents [16], [17]. We therefore hypothesized that rodents captured in the vicinity of the Interoceanic Highway of MDD would be infected with *Leishmania* (*V*.) *braziliensis*, providing further evidence to support their role as reservoirs for this pathogen in this highly prevalent region of the Peruvian Amazon. Our aim was to evaluate the most common rodent species in this area of Peru for infection with *L.* (*V.*) *braziliensis* by sampling both skin and visceral organs and screening these samples via polymerase chain reaction (PCR) assays.

## Methods

### Ethics Statement

All trapping and sampling protocols were approved by the United States Naval Medical Research Unit-6 (NAMRU-6, formerly U.S. Naval Medical Research Center, NMRCD) Institutional Animal Care and Use Committee (protocol number NMRCD10-01) and the Peruvian Ministry of Agriculture (RD 383-2009-AG-DGFFS-DGEFFS and RD 425-2010-AG-DGFFS-DGEFFS. The contract to access genetic material was granted under permit number 005-2014-MINAGRI-DGFFS-DGEFFS. Oral consent from the community leaders and the parcel owners to trap on their lands was obtained.

### Study Sites

The study sites were previously chosen as part of a larger study designed by NAMRU-6 to assess the impacts of habitat perturbation on the prevalence of pathogens in wildlife reservoirs and vectors. All sites were located adjacent to the Interoceanic Highway, which traverses Peru from the MDD Brazilian border to the Peruvian Pacific coast. At each study location, two line transects were established on either side of the road, with the exception of El Carmen where all four transects were situated on the northern side due to the inaccessibility of the southern side of the road. Transects ran approximately parallel to the Interoceanic Highway, and began at distances of 200 (near) and 1,000 (far) meters from the road.

Typically the habitat of the near transects consisted of a matrix of grassy pastures, fragmented forest patches, and small agricultural plots, or a solid stand of secondary forest. In general, the far transects ran through both primary and secondary forests, and were less disturbed (e.g. vegetation was more typical of original forest habitat) as compared to the near transects. Landscape level maps of the region's vegetation, climate, soils, geology and other Department attributes have been previously published [18], [19]. This investigation did not include an analysis of the study sites' vegetation composition.

### Field Methods

**Figure 2:**
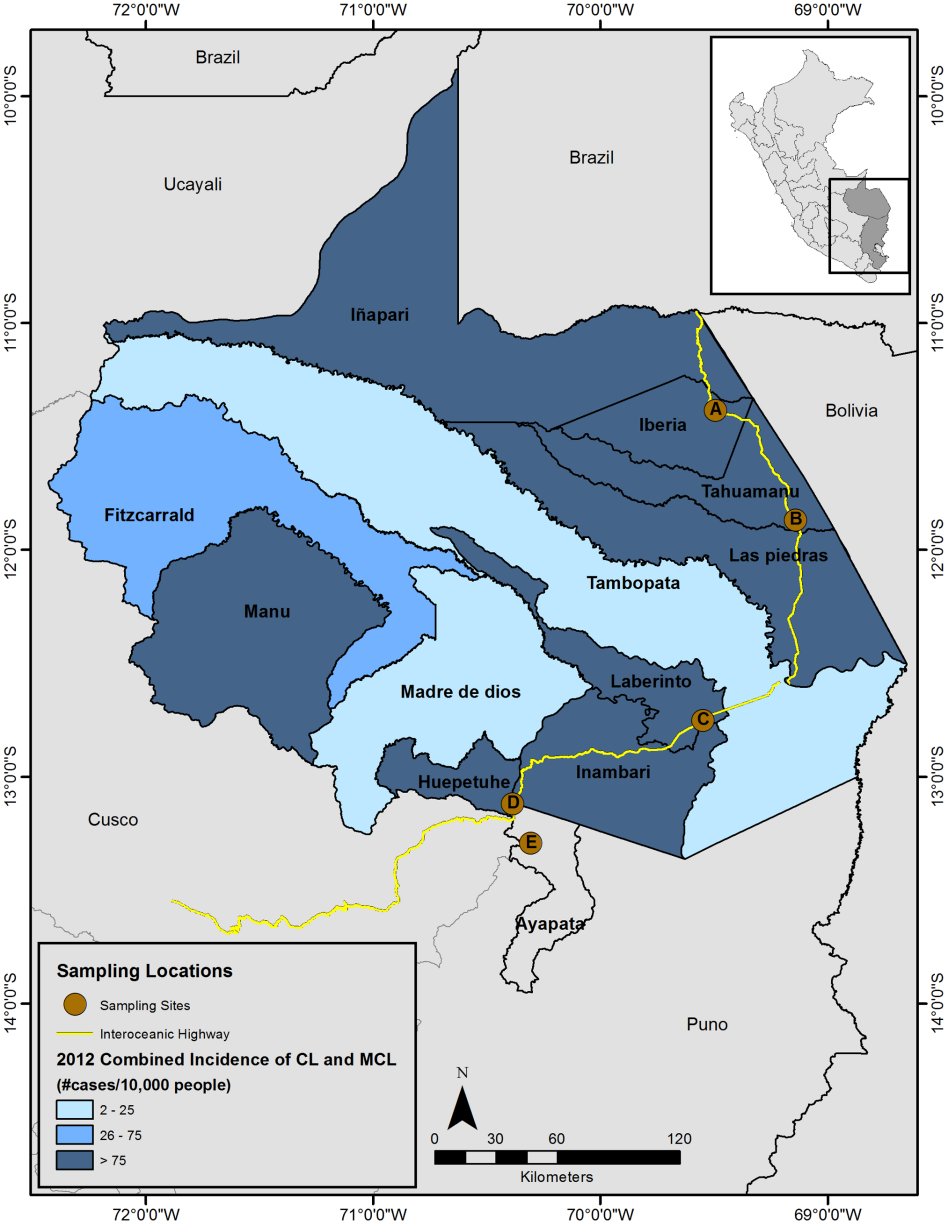
Department of Madre de Dios (Peru) human leishmaniasis incidence, by District (2012). Map of Madre de Dios with each district color-coded for the combined 2012 incidence rates of human cutaneous and mucocutaneous leishmaniasis cases, where incidence  =  [(number of probable and confirmed laboratory cases in epidemiological reporting week #51)/(2012 estimated population)]*(10,000 people). Numerator data was obtained from the Ministerio de Salud [7], [60] and population denominator data obtained from the Ministerio de Salud [61]. The Interoceanic Highway is indicated by the yellow line and the five study sites are represented by the brown dots, where A = Iberia, B = La Novia, C = Florida Baja, D = Mazuko, and E = El Carmen. El Carmen was just outside of Madre de Dios in Puno Department.

Rodents were trapped during October 2010 at two study sites, El Carmen (Puno Department) and Mazuko (MDD Department). In addition to these two locations, archived tissues were tested from rodent samples collected from three other MDD study sites: Iberia (October 2009), La Novia (October 2009), and Florida Baja (June 2010) (Figure 2).

A cluster of three Sherman traps was placed at the transect starting point and at intervals of roughly 10 meters, with the terminal location having four traps, for a total of 33 trapping points and 100 Sherman traps/line (400/study location). In addition, for the capture of larger rodents, a Tomahawk trap (model 201) was placed approximately every 30 meters (10 traps/line). Traps were re-baited daily at sundown and checked approximately 12 hours later (sunrise) on each of five consecutive nights, targeting terrestrial, nocturnal small mammals. Bait consisted of peanut butter mixed with oats, honey, raisins, sunflower seeds, and vanilla extract.

Traps containing captured animals were transported to a central processing area. Rodents were transferred from their trap to a gallon-sized zip-top clear plastic bag, through which they were anesthetized via an intraperitoneal injection of xylazine hydrochloride (Dormi-Xyl2; AgroVet Market S.A.; 20 mg/ml) and ketamine hydrochloride (Ket-A-100; Laboratorio HO Farm Agrovet Market; 100 mg/ml). The drugs were prepared by combining 0.3 ml xylazine with 3 ml ketamine in 26.7 ml of sterile water to obtain a 1∶10 drug solution. Smaller rodents (i.e. *Oligoryzomys* sp., *Neacomys* sp., etc.) received 0.3 ml of this solution, whereas 1 ml was given to larger rodents (i.e. *Proechimys* sp.). Blood was collected via intracardiac puncture from immobilized rodents, which were then euthanized by cervical dislocation. Identification of rodent species was based on the assessment of morphologic characteristics by an experienced mammalogist from the San Marcos University Natural History Museum in Lima. Complete necropsies were performed on all rodents.

Skin biopsies (ear and tail), bone marrow, and liver samples were taken at El Carmen and Mazuko. The sample size for liver tissue samples was supplemented with archived specimens from the three additional study sites previously mentioned. Liver tissues were collected into 2ml cryogenic vials with or without 100% ethanol. All vials were maintained in liquid nitrogen in the field until they could be transferred to a −80°C freezer at NAMRU-6. Tails and ears were carefully examined for active or healed lesions indicative of *Leishmania*, as described and photographically depicted in previously published papers on species other than *L*. (*V*.) *braziliensis* [16], [20]. The left ear was incised in half longitudinally from the auricular cartilage base to the tip of the ear, and the caudal half was removed. A small piece of skin was excised from the dorsal aspect of the tail, just caudal to the tail base, at the edge of the hairline. Ear and tail samples, as well as the femur for later bone marrow extraction, were each placed in separate vials containing 100% ethanol and maintained at ambient temperature.

Based on the results of our study and the binomial distribution of our data, we used R software [21] to calculate one-sided Clopper-Pearson exact 95% confidence intervals for the true prevalence values (http://cran.r-project.org/web/packages/binom/binom.pdf; “binom” package). We selected this approach for its conservative attributes, as this method generates wide confidence intervals that often have coverage probabilities larger than the specified nominal confidence level [22].

### Laboratory Methods

#### DNA extractions

All DNA extractions were performed using the DNeasy Blood and Tissue kit (Qiagen, Inc., Valencia, California, USA). Due to the inclusion of rodent hairs, which generally were not lysed by the kit reagents, slight modifications were made to the kit protocol for the skin samples. Each piece of skin was weighed on a tared microscope slide to standardize weights to ≤25 mg. Using a sterile needle, skin samples were transferred to a 1.5 ml microcentrifuge tube containing PBS 1x solution and gently swirled to remove the ethanol coating. Samples were placed on a microscope slide to which a 20 µl drop of PBS 1x was added. A razor blade was used to macerate the skin into tiny particles, and the resulting homogenate was transferred to a 1.5 ml microcentrifuge tube containing 180 µl of ATL buffer and 20 µl of Proteinase K for overnight incubation at 56°C. The following morning, 200 µl of AL buffer was added to the tube, which was then incubated at 70°C for 10 minutes. Next, 200 µl of ethanol was added, mixed thoroughly by vortexing, and centrifuged at maximum speed (14,000 rpm) for 5 minutes to form a pellet of rodent hairs. The resulting supernatant was pipetted into the kit spin column, and the remaining steps followed the kit protocol. The DNA was eluted twice in 75 µl of AE buffer, each time letting it sit at room temperature for 5–10 minutes before centrifuging, resulting in 150 µl of eluted DNA.

DNA extraction of liver and bone marrow tissues followed the kit protocol. As with the skin samples, liver samples stored in ethanol were rinsed in PBS to remove excess ethanol. The ethanol-preserved femur was split longitudinally with a razor blade and a sterile needle was used to scrape the bone marrow plug from within the cavity into a microcentrifuge tube containing ATL buffer. For all extractions, a NanoDrop spectrophotometer was used to measure the quantity of nucleic acids present in 1 µl of eluted DNA.

#### Polymerase Chain Reaction Assays

Two separate conventional PCR assays were performed to amplify sequences of the highly conserved kinetoplast minicircular DNA (kDNA), found within the mitochondria of protozoa in the taxonomical class Kinetoplastida (e.g. *Leishmania*). Each kinetoplast contains thousands of copies of kDNA, enhancing the likelihood of parasite detection and contributing to the wide popularity of primers that target kDNA in both human and animal diagnostic studies of *Leishmania* [23].

In a cross-comparison of three other primer sets used to amplify the kDNA minicircle fragment, the primers MP1-L (forward: 5′-TACTCCCCGACATGCCTCTG-3′) and MP3-H (reverse: 5′-GAACGGGGTTTCTGTATGC-3′) were found to have the greatest sensitivity as compared to other frequently used primers (e.g. B1/B2 noted in Table 1) [24]. We therefore used the MP1-L/MP3-H primers as our principle assay to amplify a 70 bp product sequence present in all species of the *Leishmania* (*Viannia*) complex [23]. The PCR reaction was performed in a volume of 20 µl, consisting of 9.2 µl of PCR grade water, 2 µl of PCR buffer (10x), 1.5 mM MgCl_2_, 125 µM dNTPs, 1 unit of Taq DNA polymerase recombinant (Invitrogen; 5U/µl), 0.5 µM of each primer, and 4 µl of template (DNA sample or positive/negative controls). The amplification thermocycler conditions were set to 5 minutes at 94°C, followed by 35 cycles each of 94°C×45 seconds, 58°C×45 seconds, and 72°C×1 minute, and a final extension cycle of 72°C for 5 minutes.

**Table 1:** Literature review of *Leishmania* (*Viannia*) *spp*. prevalence in rodents in South American countries categorized by tissue type tested.

Tissue	Rodent^1^	Country	pos/N^2^	% Positive	Species^3^	Specific assay^4^ [references in brackets]
**Blood**						
	*R. rattus*	Venezuela	3/58	5.2	*LVb*	Culture, PCR (kDNA) & RFLP [33]
	*R. rattus*	Brazil	1/61	1.6	*LVb*	PCR (kDNA; A/B) with DBH [24]
	*R. rattus*	Brazil	21/78	26.9	b-complex	nPCR (SSUrRNA; R221/R32 + R223/R333) [56]
**Bone Marrow**						
	*M. musculus*	Brazil	1/4	25	*LVb*	PCR (kDNA) & RFLP [15]
	*N. lasarius*	Brazil	2/4	50	*LVb*	PCR (kDNA) & RFLP [15]
	*R. norvegicus*	Brazil	24/78	30.8	b-complex	nPCR (SSUrRNA; R221/R32 + R223/R333) [56]
**Skin^5^**						
	*M. caliginosus*	Colombia	3/14	21.4	b-complex	PCR (kDNA; B1/B2) with DBH [57]
	*M. minutus*	Colombia	1/2	50	b-complex	PCR (kDNA; B1/B2) with DBH [57]
	*M. musculus*	Brazil	4/4	100	*LVb*	PCR (kDNA) & RFLP [15]
	*Proechimys spp*.	Brazil	1/1	100	b-complex	PCR (kDNA; B1/B2) [58]
	*P. semispinosus*	Colombia	5/33	15	*Viannia*	PCR (kDNA; B1/B2) & others [46]
	*R. norvegicus*	Brazil	8/80	10.0	b-complex	nPCR (SSUrRNA; R221/R32 + R223/R333) [56]
	*R. rattus*	Brazil	1/62	1.6	*LVb*	PCR (kDNA; A/B) with DBH [24]
	*R. rattus*	Colombia	2/4	50	b-complex	PCR (kDNA; B1/B2) with DBH [57]
**Liver**						
	*O. perenensis*	Peru	3/11	27.2	*Viannia*	PCR (kDNA; MP1-L/MP3-H) [59]
	*Proechimys spp*.	Brazil	1/1	100	b-complex	PCR (kDNA; B1/B2) [58]
**Spleen**						
	*B. lasarius*	Brazil	15/103	14.6	*LVb*	PCR (kDNA; B1/B2), MLEE & culture [12]
	*N. squamipes*	Brazil	43/153	28.1	b-complex	PCR (kDNA; B1/B2), MLEE & culture [12]
	*R. norvegicus*	Brazil	2/79	2.5	b-complex	nPCR (SSUrRNA; R221/R32 + R223/R333) [56]
	*R. rattus*	Brazil	13/81	16	b-complex	PCR (kDNA; B1/B2), MLEE & culture [12]

1 Genus information for species abbreviations in alphabetical order: B. lasarius  =  Bolomys, M. caliginosus  =  Melanomys, M. minutus  =  Microryzomys, M. musculus  =  Mus, N. lasarius  =  Necromys, N. squamipes  =  Nectomys, O. perenensis  =  Oryzoymys (since reclassified as Hylaeamys perenensis), P. semispinosus  =  Proechimys, R. norvegicus and R. rattus  =  Rattus.

2 pos/N  =  total positive/total tested

3 *LVb*  =  *Leishmania* (*Viannia*) *braziliensis*; b-complex  =  the *braziliensis* “complex” which includes the following four species: *braziliensis*, *guyanensis*, *panamensis*, and *peruviana*; *Viannia* =  identification at the subgenera level

4 PCR  =  polymerase chain reaction (gene and primer names are indicated in parentheses if reported); nPCR  =  nested PCR; RFLP  =  restriction fragment length polymorphism, DBH  =  dot-blot hybridization; MLEE  =  multi locus enzyme electrophoresis

5 Skin samples were all ear biopsies, with the exception of references #46 and #48, which were tail biopsies and unspecified skin biopsies, respectively

The second PCR assay amplified a 120 bp sequence, which in addition to species of the *L*. (*Viannia*) subgenus, also detects other *Leishmania* species such as *L*. (*L*.) *amazonensis* [25]. The primers were A (forward: 5'-SSS CCM CTA TWT TAC ACC AAC CCC-3′) and B (reverse: 5'-GGG GAG GGG CGT TCT GCG AA-3'), previously published by Oliveira [26]. The PCR reaction was performed in a volume of 25 µl, consisting of 8.55 µl of PCR grade water, 2.5 µl of PCR buffer (10x), 1.5 mM MgCl_2_, 200 µM dNTPs, 1 unit of Taq DNA polymerase recombinant (Invitrogen; 5 U/µl), 0.4 µM of each primer, and 5 µl of template. The amplification thermocycler conditions were set to 4 minutes at 94°C, followed by 35 cycles of 94°C×30 seconds, 60°C×30 seconds, and 72°C×30 seconds, and a final extension cycle of 72°C×7 minutes. This assay was restricted to a subset of the liver tissues, since we believed this tissue would be most likely to be positive for *Leishmania* species outside of the *Viannia* subgenus.

In order to verify the successful extraction of rodent DNA, a conventional PCR was run to detect a sequence of the highly conserved host housekeeping gene, interphotoreceptor retinoid-binding protein (IRBP). IRBP was previously shown to amplify across all tissue types used in this research (ear, tail, bone marrow, and liver) and all mammalian species tested, including several rodent species [27]. The IRBP PCR reaction was performed in a volume of 50 µl, consisting of 23.2 µl of PCR grade water, 5 µl of PCR buffer (10x), 1.5 mM MgCl_2_, 0.8 mM dNTPs, 1.5 units Taq DNA polymerase recombinant (Invitrogen; 5 U/µl), 0.5 µM of each primer, and 2 µl of template. The primers were IRBP-F (forward: 5′-TCCAACACCACCACTGAGATCTGGAC-3′) and IRBP-R (reverse: 5′-GTGAGGAAGAAATCGGACTGGCC-3′), previously published by Ferreira et al. [27]. The PCR amplification thermocycler conditions was set to 4 minutes at 94°C, followed by 35 cycles of 94°C×30 seconds, 57°C×30 seconds, and 72°C×1 minute, and a final extension cycle of 72°C×5 minutes. This assay, which amplified a 227 bp product sequence, was run on all bone marrow tissues and a subset of the remaining tissues.

All primers were purchased from Sigma Aldrich, and all PCR reactions were performed in the GeneAmp PCR System 9700 thermal cycler (Applied Biosystems). Products were visualized by agarose gel electrophoresis. Gels were stained with either ethidium bromide or GelRed (Phenix Research Products) and run at concentrations of 2.5%, 2%, and 1.5%, for the MP1-L/MP3-H, A/B, and IRBP assays, respectively.

#### Controls

Positive and negative controls were used in each PCR assay. The two negative controls were water and DNA extracted from ear tissue of a *Leishmania*-negative Balb-C laboratory mouse maintained at the NAMRU-6 mouse colony. The positive control DNA was derived from a culture of the *Leishmania* (*V*.) *braziliensis* reference strain MHOM/BR/84/LTB300. This DNA was diluted to a concentration of 0.2 ng/µl.

## Results

### Sampling

A total of 65 rodents were captured at El Carmen from 9-13 October 2010 and 15 were captured from Mazuko the following week (15-19 Oct). Table 2 presents the sample sizes for these two study sites by tissue type and species. *Proechimys* spp. often shed their tail upon capture, a defensive adaptation known as tail autotomy, which precluded the collection of tail tissue from some animals. A small yellowish nodule of approximately 1.5 mm in diameter was found on the tail base of a single rodent (*Hylaeamys perenensis*) at El Carmen and, in the primary author's opinion, did not match the description of *Leishmania* lesions previously described in rodents [16], [20]. A piece of the tail containing this lesion was excised for diagnostic testing. Due to time and financial constraints, we tested a random subset of approximately 50% of the archived liver samples from Florida Baja, Iberia, and La Novia. These samples closely paralleled the proportion of each species captured at each location and are listed in Table 3. The overall trap success per study site ranged from approximately 1% to 7%, where trap success was defined as the total number of captures divided by the number of trap nights (i.e. number of traps set multiplied by the number of nights trapped).

**Table 2:** Number of ear, tail, bone marrow, and liver tissues tested for *Leishmania* spp. via polymerase chain reaction shown by study site and species captured.

	El Carmen (n = 65)				Mazuko (n = 15)			
**Species** 1	Ear	Tail	BM	Liver	Ear	Tail	BM	Liver
*Hylaeamys perenensis* (n_EC_ = 31, n_MZ_ = 1)	31	27	26	31	1	1	1	1
*Neacomys spinosus* (n_EC_ = 13, n_MZ_ = 0)	13	13	12	13	0	0	0	0
*Nectomys apicalis* (n_EC_ = 1, n_MZ_ = 0)	1	0	0	0	0	0	0	0
*Oecomys bicolor* (n_EC_ = 0, n_MZ_ = 2)	0	0	0	0	2	2	2	2
*Oligoryzomys microtis* (n_EC_ = 3, n_MZ_ = 11)	2	1	1	3	10	11	11	11
*Proechimys spp.* 2 (n_EC_ = 17, n_MZ_ = 1)	17	14	17	17	1	1	1	0
**TOTAL**	**64**	**55**	**56**	**64**	**14**	**15**	**15**	**14**

1 Where n_EC_ and n_MZ_ are the total captures at El Carmen and Mazuko, respectively

2 *Proechimys spp*. included 1 *P. simonsi* at Mazuko and 6 *P. simonsi* at El Carmen. The remaining 11 at El Carmen were identified only to genus level.

**Table 3:** Liver tissue samples of rodent species from additional sites examined for *Leishmania* by polymerase chain reaction, where n_1_ is the total number captured (% of total captures) and n_2_ is the total number tested.

Study Site	La Novia		Florida Baja		Iberia	
Species	n_1_ (%)	n_2_	n_1_ (%)	n_2_	n_1_ (%)	n_2_
*Euryoryzomys nitidus*	13 (18.8)	8	6 (5.1)	3	16 (18.8)	9
*Hylaeamys perenensis*	1 (1.4)	0	3 (2.5)	2	0	0
*Neacomys spinosus*	16 (23.2)	6	0	0	5 (5.9)	1
*Necromys lenguarum*	0 (0)	0	49 (41.5)	23	0	0
*Oecomys bicolor*	4 (5.8)	1	0	0	2 (2.4)	1
*Oligoryzomys microtis*	25 (36.2)	14	58 (49.2)	35	57 (67.1)	25
*Oxymycterus inca*	1 (1.4)	0	0	0	1 (1.2)	1
*Proechimys spp.* 1	9 (13)	5	2 (1.7)	1	3 (3.5)	3
*Rattus rattus*	0 (0)	0	0	0	1 (1.2)	1
**TOTAL**	69	34	118	64	85	41

1 *Proechimys spp*. by study site consisted of: 3 *P. brevicauda* at Iberia; 6 *P. brevicauda*, 2 *P. simonsi*, and 1 *P. pattoni* at La Novia; and 1 *P. simonsi* and 1 *P. brevicauda* at Florida Baja.

For the combined total of 217 liver samples available for evaluation, 68.4% consisted of just three species: *Oligoryzomys microtis* (40.7%), *Hylaeamys perenensis* (formerly *Oryzomys perenensis*; 15.7%), and *Proechimys* spp. (12%). *O. microtis* was the most common species captured across all study sites except for El Carmen, accounting for 36%, 49%, 67%, and 73% of the total captures at La Novia, Florida Baja, Iberia, and Mazuko, respectively (Table 2). In contrast, the two most prevalent rodent species captured at El Carmen were *H*. *perenensis* (47.7%) and *Proechimys* spp. (26.2%). This study site was located in an ecoregion transitional zone between low lying amazon rainforest (“selva baja”) and high amazon rainforest (“selva alta”) and therefore its higher elevation (>500 m) likely reduced the presence of *O*. *microtis*, a species commonly found in lowland forests.

### Laboratory

*Leishmania* PCR assays for all rodent samples were negative, including the tail sample with the small nodule described above. The positive control yielded an appropriate sized PCR band in all *Leishmania* assays. Regarding assay quality assessment, the BalbC mouse housekeeping IRBP dilution trial demonstrated that the host DNA 227 bp band was present at the initial concentration of 128 ng/µl and at dilutions of 1∶10, 1∶100, and 1∶1,000, but was absent at the dilution of 1∶100,000. Despite the conclusion that amplification by PCR was effective with an extremely low DNA concentration (0.128 ng/µl), we conservatively elected to use 5 ng/µl as our cut-off value for successful DNA extraction from rodent tissues. We expected that if a rodent were infected with *Leishmania*, the parasite DNA would be at a much lower concentration than would the host DNA, and thus the inclusion of samples with DNA concentrations of <5 ng/µl could have hindered the PCR detection of *Leishmania*.

Assuming that *Leishmania* can be detected in the liver of an infected rodent, and considering that all of our samples tested negative, we concluded with 95% confidence that true *Leishmania* prevalence in the combined rodent population within our study areas was between 0–1.4% (one-sided Clopper-Pearson interval) [21]. Although results might be more meaningful if we were to evaluate each rodent species among each study site, sample sizes were not robust enough to make these comparisons. However, across all study sites combined, for those rodent species with ≥15 captures, the upper bound of the population prevalence confidence interval (CI) falls between 3.3% (sample size of 88 *O*. *microtis*) and 13.9% (sample size of 20 for *Euryoryzomys nitidus* and *Neacomys spinosus*). Likewise, when all rodent species are combined within a single study site, the CI upper bound ranges from 4.6% (El Carmen with n = 64 rodents) to 18.1% (Mazuko with n = 15 rodents). The data by species and study site is presented in Table 4.

**Table 4:** Upper bound Clopper-Pearson one-sided 95% confidence intervals for an observed *Leishmania* prevalence of 0%, evaluated by two strata: rodent species (for those species with ≥15 individuals represented) and study site.

Strata	Sample size	CP_ub_ (%)
**Rodent Species**		
*Oligoryzomys microtis*	88	3.3
*Hylaeamys perenensis*	34	8.4
*Proechimys spp.*	27	10.5
*Necromys lenguarum*	23	12.2
*Euryoryzomys nitidus*	20	13.9
*Neacomys spinosus*	20	13.9
		
**Study Site**		
El Carmen	64	4.6
Florida Baja	64	4.6
Iberia	41	7.0
La Novia	34	8.4
Mazuko	15	18.1

Calculations were performed in R using the package “binom” (http://cran.r-project.org/web/packages/binom/binom.pdf).

## Discussion

The fact that all 217 rodents examined for *Leishmania* DNA tested negative by PCR suggests that native rodents are unlikely to serve as the primary reservoir for *Leishmania* to humans in our study regions along the Transoceanic Highway in Madre de Dios (MDD). Data on human-infective *Leishmania* is available from both the National (Peruvian National Ministry of Health; MINSA) and Regional (Dirección Regional de Salud, DIRESA) government health departments. Although the number of cases fluctuates annually, in weekly epidemiological bulletins, MINSA consistently reports MDD as the Peruvian Department with the highest incidence rates [28]. In 2012, MINSA reported that MDD had a CL incidence rate six-times greater than that of the next highest Department, Amazonas (487 versus 80 cases/100,000 people) (Figure 1). Likewise, the 2012 incidence rate of MCL was more than six-times greater than the next highest Department of Cusco (45 versus 7 cases/100,000 people) (Figure 1). As shown in Figure 2, data can also be examined at the district level. All of our study sites in MDD were located in districts with comparatively high incidence rates of human leishmaniasis. Especially notable is that our study site of La Novia was situated near the border of Las Piedras and Tahuamanu, districts which in 2012 MINSA reported as having the highest incidence rates of leishmaniasis in MDD, with 208 and 175 cases/10,000 people, respectively. Therefore, from a broad-scale geographic distribution perspective, our study sites were suitable for the evaluation of *L*. (*V*.) *braziliensis* in potential wildlife reservoirs living in disturbed habitats.

However, we cannot exclude the possibility that *Leishmania* may not have been highly prevalent in the “geographic pockets” comprising our study sites at the time of sampling. Within each Department, the health system is subdivided into a network of health establishments, known as “microredes,” each of which is composed of multiple small health posts (“puestos de salud”), as well as a larger health center that serves as the head of the micored [29]. The primary level of care occurs at the health posts, where health technicians are responsible for reporting leishmaniasis cases to the centralized health centers. These data are then compiled and evaluated at both the microred and district level. As an example, the Mazuko microred, which covered solely the district of Inambari and is bisected by the Interoceanic Highway, reported 63 human cases of leishmaniasis in 2009 [6].

Research on sandfly vectors further supports our belief that *Leishmania* was likely present in our study region. In the community of Flor de Acre (Iberia district), located approximately 15 km from our Iberia study site, research in 2009 identified 33 different species of sandflies [10]. *Lutzomyia auraensis* represented 63% of the sandfly captures and several pools of this sandfly species were PCR-positive for both *L*. (*V*.) *braziliensis* and *L*. (*V*.) *lainsoni*. Moreover, positive sandflies were collected directly by human houses, proving that infected sandflies are not limited to pristine forest habitat in MDD. Furthermore, concurrent sampling of sandflies from the Mazuko, El Carmen, and Florida Baja study sites confirmed that *Leishmania* spp. is present in multiple sandfly vectors (Hugo Valdivia, personal communication, September 2013). Therefore we assume that *Leishmania* is present in our individual study sites.

In addition to geographic location, the selection of appropriate biological tissues is also important for the successful detection of pathogens in wildlife. Although in humans *L.* (*V.*) *braziliensis* is generally isolated from dermal lesions, rodents infected with this species of *Leishmania* are usually asymptomatic [30], leading to difficulty in the selection of skin biopsy sites. However, as it is thought that the sandfly vectors most readily feed upon unhaired parts of the body, such as the ear and tail, these external sites are commonly sampled for testing of cutaneous *Leishmania* species. Previous research has proven that *Leishmania* parasites can be detected from apparently normal skin (i.e. rodent ears without visible lesions) [30], [31], thus demonstrating that the pathogen may be present even in the absence of lesions. Although considered dermotrophic, studies have shown that members of the *Leishmania Viannia* subgenus may at times visceralize, depending on the host and *Leishmania* species [12], [15], [32]. Unfortunately, information on *L*. (*V*.) *braziliensis* tissue site predilection in rodents is limited to just a few studies, where the parasite has been definitively identified in the spleen [12], bone marrow [15], skin [15], and blood [33] in a few individual rodents of several species (Table 1). Although few studies have tested liver tissues of wild rodents for *L*. (*V*.) *braziliensis*, experimental studies in hamsters and wild rodents have demonstrated that this parasite will metastasize to the liver when inoculated into the skin [34], [35]. Our decision to test liver, skin, and bone marrow tissues is supported by the above and suggests that our negative laboratory results were not associated with our tissue choice selection.

The exact definition of what consititutes a reservoir has sparked scientific debates among researchers [36]–[39]. However, incidental hosts, defined as hosts that are capable of becoming infected but that are not required for disease maintenance, are most often considered irrelevant to the long-term persistence of disease. In well-studied *Leishmania* systems, reservoir hosts are usually abundant and represent a large proportion of the mammalian biomass [14]. The three most prevalent rodent species captured in our study likely fulfill the relative biomass criterion for effective reservoirs in our study areas.

Patton et al. [40] performed one of the most comprehensive small mammal surveys in the Amazon region, capturing 2,850 animals from 16 study sites along the Juruá River in the Western Amazon region of Brazil. *Oryzomys perenensis* (synonymous *H*. *perenensis*) was present in every terrestrial habitat trapped along the river and represented 16.4% of the overall species composition. In comparison, this species comprised 10.2% of the total captures in our work. *Oligoryzomys microtis* was also widely distributed in the Patton study, but had a strong preference for grassy habitats. This species has a broad geographic range across Amazonian regions of Brazil, Peru, and Bolivia, and can be found in both primary and secondary lowland forests, as well as forest edges. One notable difference between the Brazilian study and ours was with respect to the percent composition of *Proechimys* spp. and *O. microtis*. These two species comprised 12.5% and 43.8% of the total captures in our study, respectively. In contrast, in the Patton study the representation of these species was completely reversed, at 40% and 11.3%, respectively. This variation in species composition likely reflects habitat diversity, yet serves to illustrate that either species may be a dominant force in the local ecosystem's rodent biomass.

Furthermore, *Proechimys* spp. are consistently among the most frequently captured rodents in small mammal studies performed in appropriate habitat of Central and South America [41]-[43]. In the Peruvian Amazon, *Proechimys* spp. (*P*. *brevicauda* and others) accounted for 65% of 684 rodents captured over a 3-year period along the Iquitos-Nauta highway in the Department of Loreto [42]. All three *Proechimys* species captured in this study (*P*. *simonsi*, *P*. *brevicauda*, and *P*. *pattoni*) have been previously documented in the Peruvian selva baja ecoregion; however, *P*. *simonsi* has a more extensive geographic distribution, including the lower regions of higher-elevation tropical forests [44]. Thus, it makes sense that at the El Carmen study site, *P*. *simonsi* was the only *Proechimys* species identified. Members of this genus have been reported with *L*. (*V.*) *amazonensis, L (V.*) *guyanensis,* and *L.* (*V.*) *panamensis* infections under both experimental and natural conditions [41], [45]–[47]. However, we could find no confirmed reports of active infection of *L* (*V*.) *braziliensis* in *Proechimys* species.

If abundance and biomass were the sole considerations of reservoir capacity, then the three rodent species with the highest capture frequencies in our study might be likely reservoir candidates for *Leishmania* in this geographic region. However, according to Ashford [14], in well-defined *Leishmania* reservoir systems, the following additional characteristics appear to be salient features of reservoir hosts: life-span (host lives long enough to maintain infection throughout non-transmission season), response to infection (parasites localize to the skin where they are available to feeding sand flies), duration of infection (individuals remain infected without acute disease), and infection prevalence (a large proportion of individuals become infected). Our negative data indicate that these ecological requirements are not likely fulfilled by the most prevalent rodent species examined in our study.

An experimental study on *P*. *semispinosus* found that the host duration of infection was transient. Specifically, when these rodents were repeatedly infected with *L*. (*V*.) *panamensis*, they developed cutaneous lesions upon initial inoculation, but soon mounted an immune response that not only cleared infection, but also prevented lesions from forming upon subsequent inoculations [46]. Additionally, xenodiagnosis performed with two species of sand flies indicated that host-vector transmission was very poor [46]. Thus, it may be that rodents naturally infected with *L*. (*V*.) *braziliensis* also maintain a short period of infectivity, decreasing the probability that a feeding sand fly would become infected or that a PCR assay would detect a positive individual.

In the above study, a separate group of *Proechimys* was inoculated with *L*. (*Leishmania*) *chagasi,* and serology was performed to evaluate the humoral response to infection [46]. Only 60% of the ten infected animals developed anti-*Leishmania* antibodies, with levels falling below the positive threshold value within 2–4 months post-infection. Additionally, one animal with detectable *Leishmania* parasites in the spleen remained antibody-negative throughout the study. This data and the fact that *Leishmania* serology often cross-reacts with other closely related parasites (i.e. *Trypanosoma cruzi* and *T. rangeli*) [46], [48], suggests that our decision to exclude serological tests was not a limitation of our study.

Silva et al. [49] elaborate on the condition of infection prevalence, specifying that it must be higher than 20% in order for a host to be considered a primary reservoir. For those vector-borne diseases with relatively well-understood disease ecology, the pathogen prevalence in rodent reservoirs appears to support the above stated condition. For example, the tick-borne pathogens *Borrelia afzelii* and *B*. *burgdorferi*, both of which cause Lyme disease in humans, have been found at prevalences of 56.8% in bank voles and 80% in western gray squirrels, respectively [50], [51]. Similar to many species of *Leishmania*, rodents infected with *Borrelia* remain asymptomatic and testing is often via PCR of ear biopsy specimens. With respect to *L*. (*V*.) *braziliensis*, the relatively sparse data regarding parasite prevalence in potential wildlife reservoirs is suggestive that the 20% threshold criterion may be substantiated. Although the prevalence of *Leishmania* previously reported in rodents ranges greatly, it is interesting to note that in eastern Brazil, with respect to splenic tissue, the combined *Leishmania* prevalence for native rodents was 22.7% (58/256) (Table 1). In contrast, the splenic prevalence in synanthropic species (i.e. *Rattus* spp. and *Mus musculus*) was only 9.4% (15/160), perhaps reflecting the fact that these introduced species have had less time to co-evolve with the parasite. Additional research in north-eastern Brazil has suggested that the overall prevalence of *L*. (*V*.) *braziliensis* in small mammals is about 20% in this region [30]. Chaves et al. [39] cautions that due to seasonal changes in vector populations, the host prevalence can fall above or below the 20% threshold specified by Silva et. al [49]. However, the prevalence found in our study (0 to 1.7%) is so far below the suggested threshold that it is unlikely to be explained by seasonal variation in vectors. Therefore, in our study areas, the most frequently collected rodents may at most be incidental hosts, which as stated earlier, are not considered reservoirs and therefore would not constitute a major source of infection.

Although we did not detect *Leishmania* in the common rodent species captured in this study, our traps targeted primarily terrestrial rodents. We therefore cannot draw any conclusions about the reservoir potential of arboreal rodent species that may have been present in the study area but were not represented in our sample, such as bamboo rats (*Dactylomys* spp.). Nevertheless, we believe that other mammalian wildlife taxa should be more strongly considered in the transmission cycle of *L*. (*V*.) *braziliensis* in the Peruvian Amazon region. For example, research has implicated opossums as reservoirs of *L*. (*V*.) *braziliensis* [48] and sloths have been identified as reservoirs of other members of the *Leishmania Vianna* subgenus [14]. Additionally, bats have been found infected with *L*. *chagasi* and *L*. *amazonensis* [52], [53] and should not be ignored in the search for sylvatic reservoirs of *L*. (*V*.) *braziliensis*. To date, only a few studies have investigated the possible role of bats in the transmission cycle of leishmaniasis. Recently, a single study identified *L*. (*V*.) *braziliensis* from the blood, liver, and a cutaneous lesion of two bats collected in Brazil [54].

Madre de Dios is not only highly endemic for *L*. *braziliensis*, but is also rife with activities that alter the landscape, such as agriculture (croplands and cattle farming) and resource extraction industries (mining and logging). These land use practices are accompanied by the development of roads and urban areas, which create an interface among humans, domestic animals, and wildlife, especially species that are able to easily adapt to habitat modifications. The dominant rodent species captured in our study, are prime examples of such species, as our study sites were partially situated near farmlands and cattle pastures, as well as the Interoceanic Highway. Other studies have previously shown that *Oligoryzomys* spp., *Proechimys* spp., and *H*. *perenensis* are all tolerant to some level of disturbed habitat. For example, in a Brazilian study of twelve various sized fragmented forest patches located within a matrix of agriculture and cattle-raising fields, a related species, *O*. *nigripes* was consistently reported as one of the most common species captured [55]. Patton et al.[40] reported that nearly all of 321 *O*. *microtis* were captured from seasonally available grassy river edges or disturbed habitats, including old or active garden plots, pastures, and land adjacent to human dwellings. Likewise, *P*. *simonsi* and *P*. *brevicauda* are also commonly found in active and abandoned cultivated plots, as well as disturbed forests and edge habitats [40]. Patton et al. [40] noted that although *O*. *perenensis* was ubiquitous among all habitat types, it was especially tolerant of open, disturbed habitats along the river margin. Our negative results imply that although incidental infections of *L.* (*V.*) *braziliensis* may occur, the three most abundant rodent species captured in our study areas are unlikely to serve as a common source of *Leishmania* infection along the Transoceanic Highway in MDD. Therefore, the fact that these species may persist and even thrive in moderately altered landscapes, we did not find any evidence to suggest that they pose a risk for *L.* (*V.*) *braziliensis* transmission to human inhabitants in this region.

## References

[1] Desjeux P (2004) Leishmaniasis: current situation and new perspectives. Comp Immunol Microbiol Infect Dis 27: 305–318. 15225981 10.1016/j.cimid.2004.03.004

[2] Fontes CO, Carvalho MAR, Nicoli JR, Hamdan JS, Mayrink W, et al (2005) Identification and antimicrobial susceptibility of micro-organisms recovered from cutaneous lesions of human American tegumentary leishmaniasis in Minas Gerais, Brazil. J Med Microbiol 54: 1071–1076. 16192439 10.1099/jmm.0.46070-0

[3] Costa JW Jr, Milner DA Jr, Maguire JH (2003) Mucocutaneous leishmaniasis in a US citizen. Oral Surg Oral Med Oral Pathol Oral Radiol Endod 96: 573–577. 14600692 10.1016/s1079-2104(03)00299-3

[4] Lucas CM, Franke ED, Cachay MI, Tejada A, Cruz ME, et al (1998) Geographic distribution and clinical description of leishmaniasis cases in Peru. Am J Trop Med Hyg 59: 312–317. 9715953 10.4269/ajtmh.1998.59.312

[5] Urbano J, Minaya G, Sanchez-Moreno M, Gutierrez-Sanchez R, Marin C (2011) Molecular characterization and geographical distribution of leishmaniasis aethiological agents in Peru. Rev Ibero-Latinoamer Parasitol 70: 145–156.

[6] Dirección Regional de Salud Madre de Dios (2009) Análisis de la situación de salud de Madre de Dios: Documento de gestión para la toma de decisiones. Accessed March 1, 2013. http://www.minsa.gob.pe/saludmadrededios/site/ASIS%20DIRESA%20MDD%202009.pdf

[7] Perú Ministerio de Salud (2012) Casos Notificados de Leishmaniasis Cutanea Direcciones de Salud - Año 2012 SE. 51. Accessed March 1, 2013. http://www.dge.gob.pe/vigilancia/descargas/2012/51/LEISHMANIASIS%20CUTANEA.pdf

[8] Instituto Nacional de Estadística e Informática (2012) Perú: Estimaciones y Proyecciones de Población Total por Sexo de las Principales Ciudades, 2000–2015. Boletín Especial N° 23. Accessed March 1, 2013. http://www.inei.gob.pe/biblioineipub/bancopub/Est/Lib1020/Libro.pdf

[9] Rojas-Jaimes J (2012) Leishmaniasis: una enfermedad desatendida. Un acercamiento a la realidad en Huepetuhe, Madre de Dios. Revista Peruana de Epidemiología 16: 4pp.

[10] Valdivia HO, De Los Santos MB, Fernandez R, Christian Baldeviano G, Zorrilla VO, et al (2012) Natural *Leishmania* Infection of *Lutzomyia auraensis* in Madre de Dios, Peru, Detected by a Fluorescence Resonance Energy Transfer-Based Real-Time Polymerase Chain Reaction. Amer J Trop Med Hyg 87: 511–523. 22802444 10.4269/ajtmh.2012.11-0708 PMC3435357

[11] Aguilar CM, Rangel EF, Garcia L, Fernandez E, Momen H, et al (1989) Zoonotic cutaneous leishmaniasis due to *Leishmania braziliensis* associated with domestic animals in Venezuela and Brazil. Mem Inst Oswaldo Cruz 84: 19–28. 2319948 10.1590/s0074-02761989000100005

[12] Brandão-Filho SP, Brito ME, Carvalho FG, Ishikaw EA, Cupolillo E, et al (2003) Wild and synanthropic hosts of *Leishmania* (*Viannia*) *braziliensis* in the endemic cutaneous leishmaniasis locality of Amaraji, Pernambuco State, Brazil. Trans R Soc Trop Med Hyg 97: 291–296. 15228244 10.1016/s0035-9203(03)90146-5

[13] Cupolillo E, Brahim LR, Toaldo CB, de Oliveira-Neto MP, de Brito ME, et al (2003) Genetic polymorphism and molecular epidemiology of *Leishmania* (*Viannia*) *braziliensis* from different hosts and geographic areas in Brazil. J Clin Microbiol 41: 3126–3132. 12843052 10.1128/JCM.41.7.3126-3132.2003 PMC165365

[14] Ashford RW (1996) Leishmaniasis Reservoirs and Their Significance in Control. Clin Dermatol 14: 523. 8889331 10.1016/0738-081x(96)00041-7

[15] Tavares de Freitas TP, D'Andrea PS, Jesus de Paula DA, Nakazato L, Dutra V, et al (2012) Natural Infection of *Leishmania* (*Viannia*) *braziliensis* in *Mus musculus* Captured in Mato Grosso, Brazil. Vector Borne Zoonotic Dis 12: 81–83. 21923255 10.1089/vbz.2010.0268

[16] Kerr SF, Emmons LH, Melby PC, Liu C, Perez LE, et al (2006) *Leishmania amazonensis* infections in *Oryzomys acritus* and *Oryzomys nitidus* from Bolivia. Amer J Trop Med Hyg 75: 1069–1073. 17172367

[17] Kerr SF, McHugh CP, Merkelz R (1999) Short report: a focus of *Leishmania mexicana* near Tucson, Arizona. Amer J Trop Med Hyg 61: 378–379. 10497974 10.4269/ajtmh.1999.61.378

[18] República del Peru Ministerio de Agricultura (1994) Zonificación ecológica-económica Yaco-Iñapari e Iberia-Iñapari, Madre de Dios: Diagnostico Ambiental Iberia-Iñapari. Instituto Nacional de Recursos Naturales (INRENA). Lima, Peru. pp. 368.

[19] Madre de Dios, Camino al desarollo sostenible: Propuesta de zonificación ecológica econónmica como base para el ordenamiento territorial. Instituto de Investigaciones de la Amazona Peruana. Iquitos, Peru. pp. 137.

[20] Chable-Santos JB, Van Wynsberghe NR, Canto-Lara SB, Andrade-Narvaez FJ (1995) Isolation of *Leishmania* (*L*.) *mexicana* from wild rodents and their possible role in the transmission of localized cutaneous leishmaniasis in the state of Campeche, Mexico. Amer J Trop Med Hyg 53: 141. 7677214 10.4269/ajtmh.1995.53.141

[21] R Development Core Team (2011). R: A language and environment for statistical computing. R. Foundation for Statistical Computing, Vienna, Austria. ISBN 3-900051-07-0, URL http://www.R-project.org

[22] Brown L, Cai T, DasGupta A (2001) Interval Estimation for a Binomial Proportion. Stat Sci 16: 101–133.

[23] Lopez M, Inga R, Cangalaya M, Echevarria J, Llanos-Cuentas A, et al (1993) Diagnosis of *Leishmania* using the polymerase chain reaction: a simplified procedure for field work. Am J Trop Med Hyg 49: 348–356. 8396860 10.4269/ajtmh.1993.49.348

[24] Molina de Oliveira D, Valdrinez M, Lonardoni C, Teodoro U, Verzignassi Silveira TG (2011) Comparison of different primes for PCR-based diagnosis of cutaneous leishmaniasis. Braz J Infec Dis 15: 204–210. 21670918 10.1016/s1413-8670(11)70176-3

[25] Quaresma PF, Fonseca Murta SM, de Castro Ferreira E, da Rocha-Lima ACVM, Prates Xavier AA, et al (2009) Molecular diagnosis of canine visceral leishmaniasis: Identification of *Leishmania* species by PCR-RFLP and quantification of parasite DNA by real-time PCR. Acta Trop 111: 289–294. 19467216 10.1016/j.actatropica.2009.05.008

[26] Oliveira FS, Pirmez C, Pires MQ, Brazil RP, Pacheco RS (2005) PCR-based diagnosis for detection of *Leishmania* in skin and blood of rodents from an endemic area of cutaneous and visceral leishmaniasis in Brazil. Vet Parasitol 129: 219–227. 15845276 10.1016/j.vetpar.2005.01.005

[27] Ferreira EC, Gontijo CM, Cruz I, Melo MN, Silva AM (2010) Alternative PCR protocol using a single primer set for assessing DNA quality in several tissues from a large variety of mammalian species living in areas endemic for leishmaniasis. Mem Inst Oswaldo Cruz 105: 895–898. 21120359 10.1590/s0074-02762010000700009

[28] Perú Ministerio de Salud (2009, 2010, 2011) Sala de Situación de Salud, Perú. Dirreción General de Epidemilogía. Accessed March 1, 2013. http://www.dge.gob.pe/salasit.php

[29] Guthmann J-P, Arlt D, Garcia LML, Rosales M, Sanchez JdJ, et al (2005) Control of mucocutaneous leishmaniasis, a neglected disease: results of a control programme in Satipo Province, Peru. Tropical Medicine & International Health 10: 856–862. 16135192 10.1111/j.1365-3156.2005.01460.x

[30] Lima B, Dantas-Torres F, de Carvalho M, Marinho-Junior J, EL dA, et al (2013) Small mammals as hosts of *Leishmania* spp. in a highly endemic area for zoonotic leishmaniasis in north-eastern Brazil. Trans R Soc Trop Med Hyg 107: 592–597. 23868744 10.1093/trstmh/trt062

[31] Lainson R, Shaw JJ (1968) Leishmaniasis in Brazil: I. Observations on enzootic rodent leishmaniasis—incrimination of *Lutzomyia flaviscutellata* (mangabeira) as the vector in the lower amazonian basin. Trans R Soc Trop Med Hyg 62: 385–395. 5659232 10.1016/0035-9203(68)90090-4

[32] Reithinger R, Lambson BE, Barker DC, Davies CR (2000) Use of PCR to detect *Leishmania* (*Viannia*) spp. in dog blood and bone marrow. J Clin Microbiol 38: 748–751. 10655379 10.1128/jcm.38.2.748-751.2000 PMC86194

[33] De Lima H, De Guglielmo Z, Rodriguez A, Convit J, Rodriguez N (2002) Cotton rats (*Sigmodon hispidus*) and black rats (*Rattus rattus*) as possible reservoirs of *Leishmania* spp. in Lara State, Venezuela. Mem Inst Oswaldo Cruz 97: 169–174. 12016437 10.1590/s0074-02762002000200004

[34] Almeida MC, Cuba-Cuba CA, Moraes MAP, Miles MA (1996) Dissemination of *Leishmania* (*Viannia*) *braziliensis* . J Comp Path 115: 311–316. 8923241 10.1016/s0021-9975(96)80088-0

[35] Rodriguez Roque AL, Cupolillo E, Marchevsky RS, Jansen AM (2010) *Thrichomys laurentius* (Rodentia; Echimyidae) as a Putative Reservoir of *Leishmania infantum* and *L* *braziliensis*: Patterns of Experimental Infection. PLoS Negl Trop Dis: Public Library of Science. pp. e589. 10.1371/journal.pntd.0000589 PMC2814861 20126407

[36] Ashford RW (1997) What it takes to be a reservoir host. Belg J Zool 127: 85.

[37] Ashford RW (2003) When is a reservoir not a reservoir? Emerging Infect Dis 9: 1495–1496. 14725261 10.3201/eid0911.030088 PMC3035539

[38] Haydon DT, Cleaveland S, Taylor LH, Laurenson MK (2002) Identifying reservoirs of infection: a conceptual and practical challenge. Emerging Infect Dis 8: 1468–1473. 12498665 10.3201/eid0812.010317 PMC2738515

[39] Chaves LF, Hernandez MJ, Dobson AP, Pascual M (2007) Sources and sinks: revisiting the criteria for identifying reservoirs for American cutaneous leishmaniasis. Trends Parasitol 23: 311–316. 17524806 10.1016/j.pt.2007.05.003

[40] Patton JL, Da Silva MNF, Malcolm JR (2000) Mammals of the Rio Jurua and the evolutionary and ecological diversification of Amazonia. Bull Am Mus Nat Hist: 3–306.

[41] Dedet JP, Gay F, Chatenay G (1989) Isolation of *Leishmania* species from wild mammals in French Guiana. Trans R Soc Trop Med Hyg 83: 613–615. 2617621 10.1016/0035-9203(89)90374-x

[42] Diaz MM, Nava S, Alejandro Guglielmone A (2009) The parasitism of *Ixodes luciae* (Acari: Ixodidae) on marsupials and rodents in Peruvian Amazon. Acta Amazonica 39: 997–1002.

[43] Barrera R, Ferro C, Navarro JC, Freier J, Liria J, et al (2002) Contrasting sylvatic foci of Venezuelan equine encephalitis virus in northern South America. Am J Trop Med Hyg 67: 324–334. 12408676 10.4269/ajtmh.2002.67.324

[44] Pacheco V, Cadenillas R, Salas E, Tello C, Zeballos H (2009) Diversidad y endemismo de los mamíferos del Perú. Rev Peru Biol.

[45] Thatcher VE, Eisenmann C, Hertig M (1965) Experimental inoculation of Panamanian mammals with *Leishmania braziliensis* . J Parasitol 51: 842–844. 5857288

[46] Travi BL, Arteaga LT, Leon AP, Adler GH (2002) Susceptibility of Spiny Rats (*Proechimys semispinosus*) to *Leishmania* (*Viannia*) *panamensis* and *Leishmania* (*Leishmania*) *chagasi* . Mem Inst Oswaldo Cruz 97: 887–892. 12386716 10.1590/s0074-02762002000600025

[47] Arias JR, Naiff RD, Miles MA, de Souza AA (1981) The opossum, *Didelphis marsupialis* (Marsupialia: Didelphidae), as a reservoir host of *Leishmania braziliensis guyanensis* in the Amazon Basin of Brazil. Trans R Soc Trop Med Hyg 75: 537–541. 7324129 10.1016/0035-9203(81)90194-2

[48] Schallig HDFH, Da Silva ES, Van Der Meide WF, Schoone GJ, Gontijo CMF (2007) *Didelphis marsupialis* (common opossum): a potential reservoir host for zoonotic leishmaniasis in the metropolitan region of Belo Horizonte (Minas Gerais, Brazil). Vector Borne Zoonotic Dis 7: 387–393. 17767408 10.1089/vbz.2006.0651

[49] Silva E, Gontijo C, Melo M (2005) Contribution of molecular techniques to the epidemiology of neotropical *Leishmania* species. Trends Parasitol 21: 550–552. 16226490 10.1016/j.pt.2005.09.008

[50] Vaumourin E, Gasqui P, Buffet JP, Chapuis JL, Pisanu B, et al (2013) A probabilistic model in cross-sectional studies for identifying interactions between two persistent vector-borne pathogens in reservoir populations. PLoS One 8: e66167. 23840418 10.1371/journal.pone.0066167 PMC3688727

[51] Lane RS, Mun J, Eisen RJ, Eisen L (2005) Western Gray Squirrel (Rodentia: Sciuridae): A Primary Reservoir Host of Borrelia burgdorferi in Californian Oak Woodlands? J Med Entomol 42: 388–396. 15962792 10.1093/jmedent/42.3.388

[52] De Lima H, Rodriguez N, Barrios MA, Avila A, Canizales I, et al (2008) Isolation and molecular identification of *Leishmania chagasi* from a bat (*Carollia perspicillata*) in northeastern Venezuela. Mem Inst Oswaldo Cruz 103: 412–414. 18661000 10.1590/s0074-02762008000400018

[53] Savani ESMM, Fernandes de Almeida M, de Oliveira Camargo MCG, D'Auria SRN, Silva MMS, et al (2010) Detection of *Leishmania* (*Leishmania*) *amazonensis* and *Leishmania* (*Leishmania*) *infantum chagasi* in Brazilian bats. Vet Parasitol 168: 5–10. 19939568 10.1016/j.vetpar.2009.10.019

[54] Shapiro JT, da Costa Lima Junior MS, Cavalheiros Dorval ME, de Oliveira França A, de Fatima Cepa Matos M, et al (2013) First record of *Leishmania braziliensis* presence detected in bats, Mato Grosso do Sul, southwest Brazil. Acta Trop 128: 171–174. 23886850 10.1016/j.actatropica.2013.07.004

[55] Vaz VC, D'Andrea PS, Jansen AM (2007) Effects of habitat fragmentation on wild mammal infection by *Trypanosoma cruzi* . Parasitol 134: 1785–1793. 10.1017/S003118200700323X 17651530

[56] Marcelino AP, Ferreira EC, Avendanha JS, Costa CF, Chiarelli D, et al (2011) Molecular detection of *Leishmania braziliensis* in *Rattus norvegicus* in an area endemic for cutaneous leishmaniasis in Brazil. Vet Parasitol 183: 54–58. 21767914 10.1016/j.vetpar.2011.06.019

[57] Alexander B, Lozano C, Barker DC, McCann SHE, Adler GH (1998) Detection of *Leishmania* (*Viannia*) *braziliensis* complex in wild mammals from Colombian coffee plantations by PCR and DNA hybridization. Acta Trop 69: 41–50. 9588240 10.1016/s0001-706x(97)00114-9

[58] De Bruijn MHL, Barker DC (1992) Diagnosis of New World leishmaniasis: Specific detection of species of the *Leishmania braziliensis* complex by amplification of kinetoplast DNA. Acta Trop 52: 45–58. 1359760 10.1016/0001-706x(92)90006-j

[59] Quispe Flórez MM (2002) Búsqueda de roedores posibles reservorios de *Leishmania* en el distrito de Echarati, Provincia de la Convención. Cusco: Universidad Nacional de San Antonio Abad del Cusco. 97 p.

[60] Perú Ministerio de Salud (2012) Casos Notificados de Leishmanisis Mucocutanea: Direcciones de Salud - Año 2012 SE. 51. Accessed March 1, 2013. http://www.dge.gob.pe/vigilancia/descargas/2012/51/LEISHMANIASIS%20MUCOCUTANEA.pdf

[61] Perú Ministerio de Salud (2012) Poblacíon Estimada por Edades Puntuales, Grupos Quinquenales y Grupos Especiales Según Departamento, Provinicia y Distrito Perú: 2012. Accessed March 1, 2013.

